# CML/RAGE Signal Bridges a Common Pathogenesis Between Atherosclerosis and Non-alcoholic Fatty Liver

**DOI:** 10.3389/fmed.2020.583943

**Published:** 2020-11-06

**Authors:** Qiwen Pang, Zhen Sun, Chen Shao, Honghua Cai, Zhengyang Bao, Lin Wang, Lihua Li, Lele Jing, Lili Zhang, Zhongqun Wang

**Affiliations:** ^1^Department of Cardiology, Affiliated Hospital of Jiangsu University, Zhenjiang, China; ^2^Department of Burn Surgery, Affiliated Hospital of Jiangsu University, Zhenjiang, China; ^3^Department of Internal Medicine, Affiliated Hospital of Wuxi Maternity and Child Health of Nanjing Medical University, Wuxi, China; ^4^Department of Pathology, Affiliated Hospital of Jiangsu University, Zhenjiang, China

**Keywords:** atherosclerosis (AS), non-alcoholic fatty liver disease (NAFLD), Nε-carboxymethyllysine (CML), advanced glycosylation end-product receptor (RAGE), pro-inflammatory

## Abstract

Non-alcoholic fatty liver disease (NAFLD) has become a common chronic disease in the world. NAFLD is not only a simple intrahepatic lesion, but also affects the occurrence of a variety of extrahepatic complications. In particular, cardiovascular complications are particularly serious, which is the main cause of death in patients with NAFLD. To study the relationship between NAFLD and AS may be a new way to improve the quality of life in patients with NAFLD. As we all known, inflammatory response plays an important role in the occurrence and development of NAFLD and AS. In this study, we found that the accumulation of Nε-carboxymethyllysine (CML) in the liver leads to hepatic steatosis. CML can induce the expression of interleukin (IL-1β), interleukin (IL-6), tumor necrosis factor (TNF-α), C-reactionprotein (CRP) by binding with advanced glycosylation end-product receptor (RAGE) and accelerate the development of AS. After silencing RAGE expression, the expression of pro-inflammatory cytokines was inhibited and liver and aorta pathological changes were relieved. In conclusion, CML/RAGE signal promotes the progression of non-alcoholic fatty liver disease and atherosclerosis. We hope to provide new ideas for the study of liver vascular dialogue in multi organ communication.

## Introduction

With economic development, improvement in living standards, the prevalence of a high-fat, high-calorie diet, the acceleration of life and the prevalence of a lifestyle of less movement and more sitting, the prevalence of non-alcoholic fatty liver disease (NAFLD) is increasing (1, 2). NAFLD refers to excessive fat deposition in the liver in the absence of ethanol and other clear causes. The main characteristics of this disease are accumulation and diffuse fatty degeneration of hepatocytes (3, 4). NAFLD is the most common chronic liver disease (4, 5), and NAFLD has become a worldwide public health problem that endangers human health. According to statistics, the global prevalence of NAFLD has reached 25.2% (6). NAFLD has no obvious symptoms in the early stage, and liver fat infiltration of 30% or more can be diagnosed by liver ultrasound (7–9). As NAFLD is difficult to diagnose and its prevalence is likely to be underestimated.

As early as 1950, researchers found that NAFLD is associated with Atherosclerosis (AS) (10). NAFLD and AS often share common pathogenic factors. Exploring its common pathogenic factors not only inhibits the occurrence of NAFLD, it may even protect NAFLD patients from AS (11, 12).

At present, the “second strike” hypothesis is widely accepted regarding the pathogenesis of NAFLD, which is mainly caused by excessive lipid accumulation in the liver and oxidative stress caused by lipid peroxidation (13). Lipid peroxidation induces inflammation in the liver and promotes the formation of advanced glycation end products (AGEs) (14–17). Once AGEs are formed, they are difficult to degrade and accumulate in the body with age. Nε-carboxymethyllysine (CML) is the most important active center of AGEs (18). By binding with its receptor for AGEs (RAGE), it destroys cell antioxidant defense and the production of ROS (19). CML activate the receptor RAGE to cause cell activation and increase the production of pro-inflammatory cytokines (such as IL-1β, IL-6, TNF-α, CRP), leading to the occurrence of various diseases (20–22). Our previous research found that CML/RAGE signaling plays an important role in the development of AS (23). CML-RAGE interaction can change the role of the endothelial barrier, increase the permeability of endothelial cells, and destroy the normal function of vascular endothelial cells (24). CML-RAGE also promotes smooth muscle cells to take up excessive cholesterol and induces the formation of vascular smooth muscle cell-derived foam cells (25). Eventually promote the formation and progression of AS.

Liver is an important metabolic center of AGEs in the body (26). Existing research shows that AGEs research has found that AGEs have a significant effect on liver cells, such as promoting the release of pro-inflammatory cytokines and participating in the formation of liver fibrosis. We hypothesized that CML/RAGE signaling may be a common risk factor for the development of NAFLD and AS.

In this study, we collected liver biopsies from atherosclerotic patients, and used an *in vivo* model to explored the role of CML/RAGE in the development of NAFLD and AS.

## Methods

### Patients

Liver biopsy specimens of 80 individuals undergoing liver biopsy were collected from the affiliated hospital of Jiangsu University (The clinical baseline data in Supplementary Table 1). Liver biopsies were fixed in formalin and embedded in paraffin. We divided the patients into 4 groups according to the degree of steatosis:control group (no steatosis or steatotic liver cells <5%) the low steatosis group (steatotic liver cells 5–33%); the moderate steatosis group (steatotic liver cells 33–66%); and the severe steatosis group (steatotic liver cells> 66%).

### Inclusion Criteria

1. Age: 40–75 years;
2. Voluntarily undergo liver biopsy and sign the consent form.

### Exclusion Criteria

1. Drinking history (daily alcohol intake: female <20 g/d, male <30 g/d);
2. Viral hepatitis, drug-induced liver disease, total parenteral nutrition, hepatolenticular degeneration, autoimmune liver disease and other specific diseases that can cause fatty liver;
3. Excluded drugs (amiodarone, tamoxifen, sodium valproate, glucocorticoid, methotrexate, etc.), total parenteral nutrition, inflammatory bowel disease, hypothyroidism, etc. Special conditions of fatty liver;
4. History of infection or tissue damage in the last 1 month;
5. A history of malignant tumors or autoimmune diseases; and a history of liver transplant surgery.

## Animals

Six-week-old ApoE^−/−^ mice on a C57BL/6J background were purchased fromurchased from Cavens (Changzhou, China), Animals are kept in the barrier system of Jiangsu University Laboratory Animal Research Center. All animal experiments were approved by Institutional Animal Care and Use Committee of Jiangsu University. 10^12^ DNAse-resistant particles of adeno-associated viral (AAV) vectors consisting of AAV-shscramble (as a control group), AAV-shRAGE (Han HengBiological Technology Co., Ltd. Shanghai China) solution was injected into mice via the tail vein. 3 weeks after AAV injection, the high-fat diet (HFD) was introduced. Inject CML (10 mg/kg/day) into the tail vein of mice. The mice were randomly divided into 5 groups: control group (normal diet); model group (high-fat diet); CML group (high-fat diet+CML 10 mg/kg/d), AAV-shscramble group (high-fat diet +CML 10 mg/kg/d+AAV-shscramble); AAV-shRAGE group (high-fat diet +CML 10 mg/kg/d+AAV-shRAGE). Observe the pathological changes of the liver and aorta at 4, 8, 16 weeks after CML injection. Blood samples were collected through the tail vein at 16 weeks. After the mice were euthanized, the abdominal cavity and chest cavity were opened, liver tissues were removed, and the liver wet weight was accurately weighed to calculate the liver index. The liver index was calculated as follows: liver index = wet liver weight (g)/body weight (g).

### Biochemical Analysis

The levels of total cholesterol (TC), triglyceride (TG), low-density lipoprotein (LDL-C), high-density lipoprotein (HDL-C), alanine aminotransferase (ALT), aspartate aminotransferase (AST) were measured by automatic biochemical instrument.

### Immunohistochemical Staining

Paraffin sections were dewaxed, rehydrated and boiled in citric acid buffer for 10 min for antigen repair. The sections were blocked with 5% goat serum at room temperature for 1 h. Human liver samples were incubated with CML antibody (Abcam, 1:200) and RAGE antibody (Abcam, 1:200). ApoE^−/−^ mice liver and aorta samples were incubated with RAGE antibody (Abcam, 1:200) overnight at 4°C. Then, we used a rabbit and mouse HRP kit (Conway, China Century Biotechnology Co., Ltd.) sample was photographed under a microscope.

### H&E Staining

The aorta and liver tissue were fixed in 4% formaldehyde buffer and then embedded in paraffin. Paraffin embedded tissue was cut into 5 μm thick sections and stained with hematoxylin and eosin. The stained samples were photographed under a microscope.

### Oil Red O Staining

Frozen sections of mice liver were fixed with 4% paraformaldehyde for 1 h and washed with isopropanol 3 times (15 s each time). Then, the slices were dyed in oil red O working solution (3 oil red O stock solution: 2 distilled solution) for 30 min and washed in isopropanol 3 times. The stained samples were photographed under a microscope.

### Detection of Gene Expression by Real-time Quantitative PCR

Total RNA was extracted from the mice liver and aorta using TRIzol reagent (Invitrogen, Carlsbad, CA, USA). The RNA concentration was determined by measuring the optical density at 260 and 280 nm. Then, the RNA was reverse transcribed into cDNA. Real-time PCR was performed using primers for mice IL-1β, IL-6, TNF-α, CRP, and β-actin (synthesized by Sangon). The primer sequences are detailed in Supplementary Table 1. The RT-PCR reaction conditions were as follows: 94°C for 1 min, followed by 33 cycles at 94°C for 30 s, 63°C for 30 s, and 72°C for 1 min, and a final extension at 72°C for 7 min.

### Statistical Analysis

All data are expressed as mean ± SD.SPSS 25.0 was used for analysis. The multiple groups were compared using a one-way analysis of variance and between two groups by Student's *t*-test analysis. *p* < 0.05 was considered statistically significant.

## Results

### CML Accumulation in Liver Tissue of Patients With Non-alcoholic Fatty Liver

**Figure 1:**
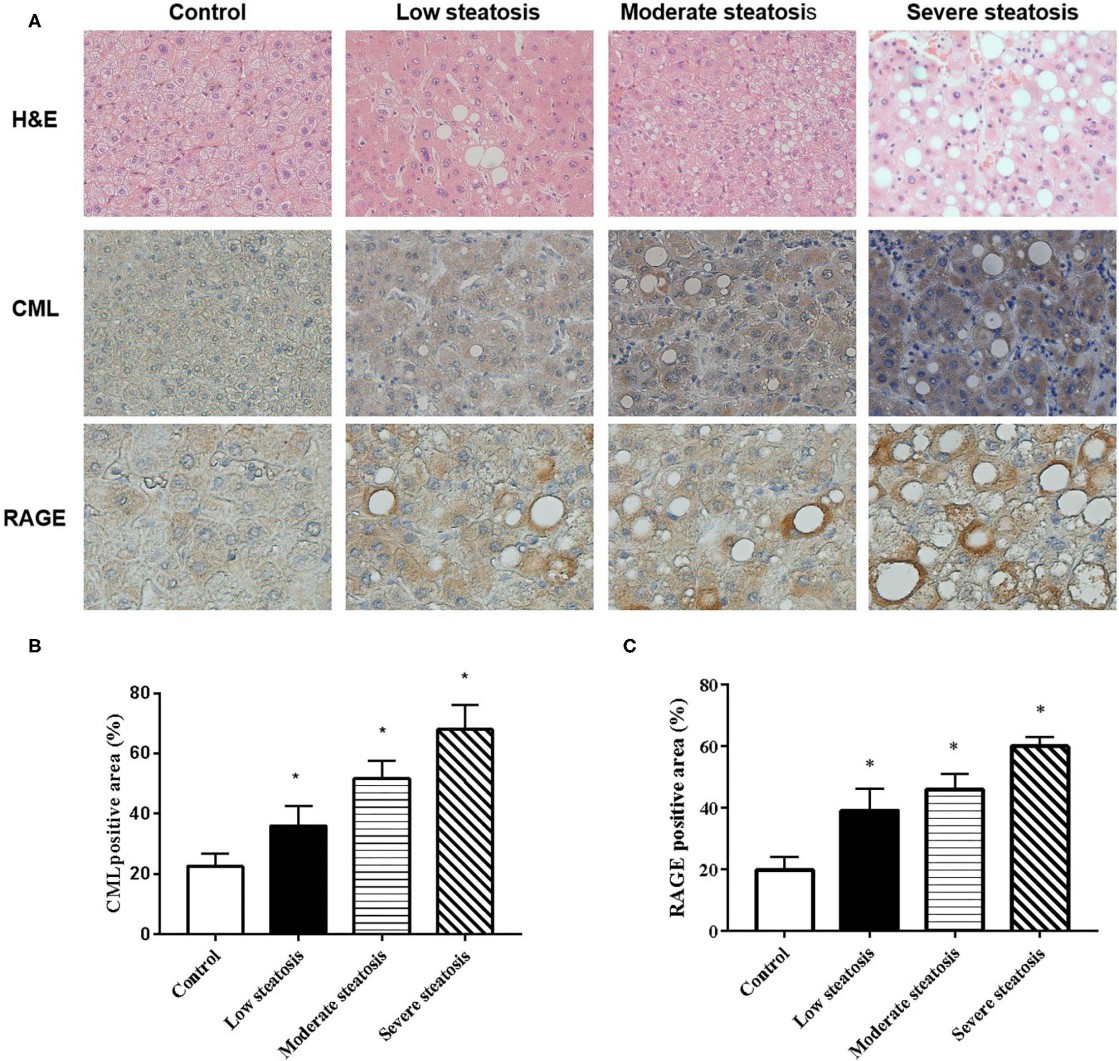
CML accumulation in liver tissue of patients with non-alcoholic fatty liver **(A)**. First row: Liver biopsy H&E (×200). Second and third row: immunohistochemistry shows the expression of CML (×200) and RAGE (×400) **(B)**. Percentage of CML-positive immunohistochemical staining area **(C)**. Percentage of RAGE-positive immunohistochemical staining area. The proportion of the positive area to the total liver area was calculated by ImageJ. All data are expressed as the means ± SEM. *P < 0.05, compared to control group.

We performed liver biopsies on 80 individuals in the affiliated hospital of Jiangsu University. The degree of fatty degeneration of the liver was divided into four groups according to H&E staining (Figure 1A). To investigate whether CML accumulates in the liver, we performed immunohistochemical staining. We observed no obvious CML staining in the liver tissue of the control group (Figures 1A,B). With steatosis of the liver, CML staining gradually increased. We detected the expression of the CML receptor RAGE by immunohistochemistry and found that RAGE was significantly expressed on the steatotic liver cell membrane (Figures 1A,C). We hypothesize that liver steatosis may be related to CML accumulation and CML may play a role in inducing liver inflammation by binding with RAGE.

### CML Promotes High Fat-Induced Liver Steatosis and Intravascular Plaque Formation in ApoE^-/-^ Mice

**Figure 2:**
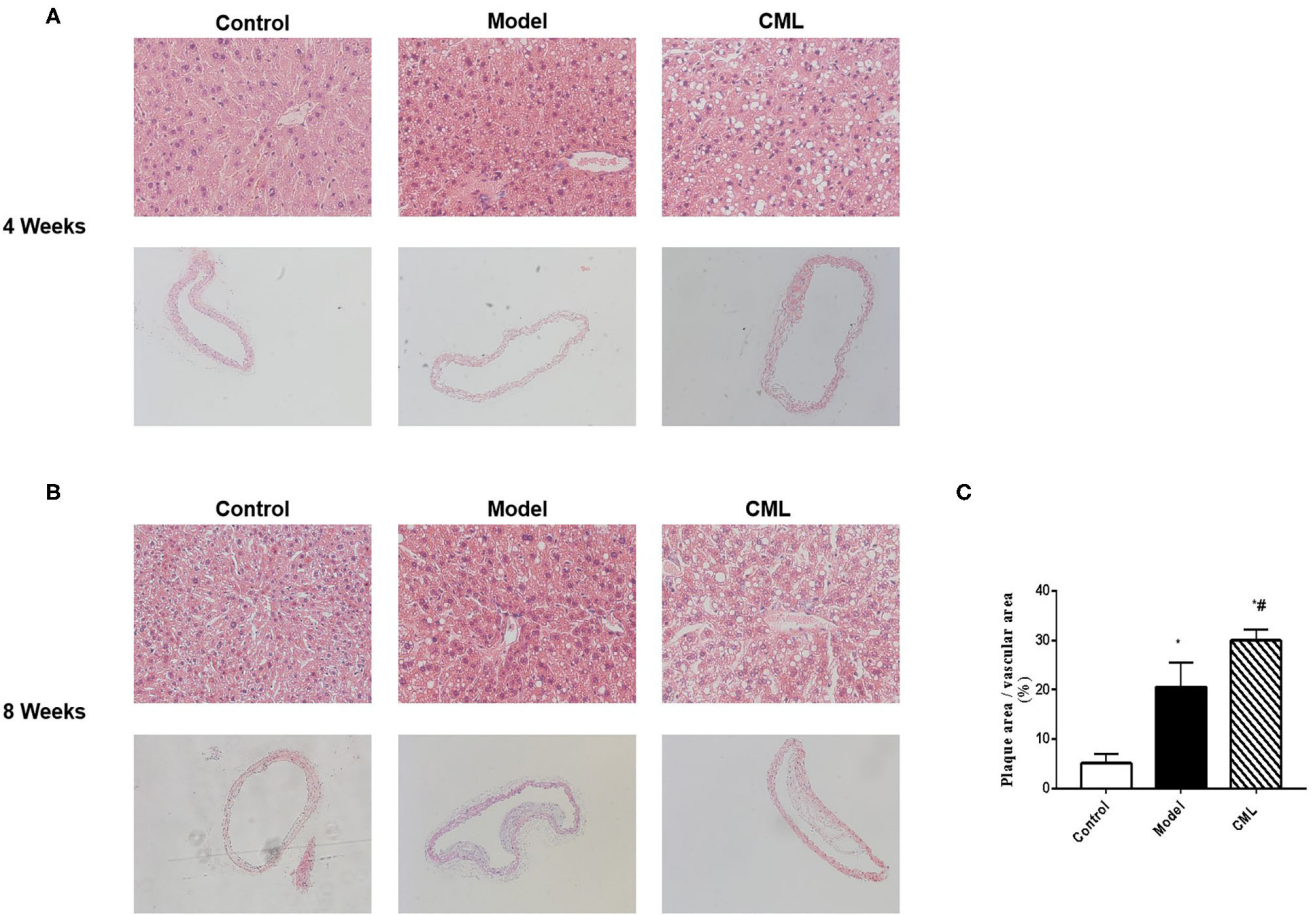
CML promotes high fat-induced liver steatosis and intravascular plaque formation in ApoE^−/−^ mice **(A,B)**. At the time of 4, 8 weeks, ApoE^−/−^ mice liver (×200) and aorta H&E staining (×100) **(C)**. Image J analyzes plaque area.*P < 0.05, compared to control group; ^#^*P* < 0.05, compared to model group.

The pathological changes of liver and aorta of ApoE^−/−^ mice were detected by H&E staining in 4 and 8 weeks (Figure 2). At the time of 4 weeks, the hepatocytes were arranged in a radial pattern, hepatocytes were uniform in size, the nucleus was centrally distributed, thickness of the vascular intima was uniform, and the endoplasm was neatly arranged in the control group. In the model group, hepatocytes showed balloon-like degeneration, hepatocytes were uneven in size, the nucleus shifted to one side, and the vascular endoplasmic arrangement was disordered, the thickness of intima and media was uneven. In the CML group, hepatocytes were larger in size, with obvious balloon-like degeneration of hepatocytes and the thickness of intima and media increased, and a large number of monocytes gathered (Figure 2A). At the time of 8 weeks (Figures 2B,C), the fatty degeneration of liver cells in mice was further aggravated, and foam cells and fibrous plaques can be seen under the vascular intima, which protrude into the lumen, and the basement membrane is destroyed. After CML intervention, the mice liver steatosis and severe changes in aortic plaque compared with the model group.

**Figure 3:**
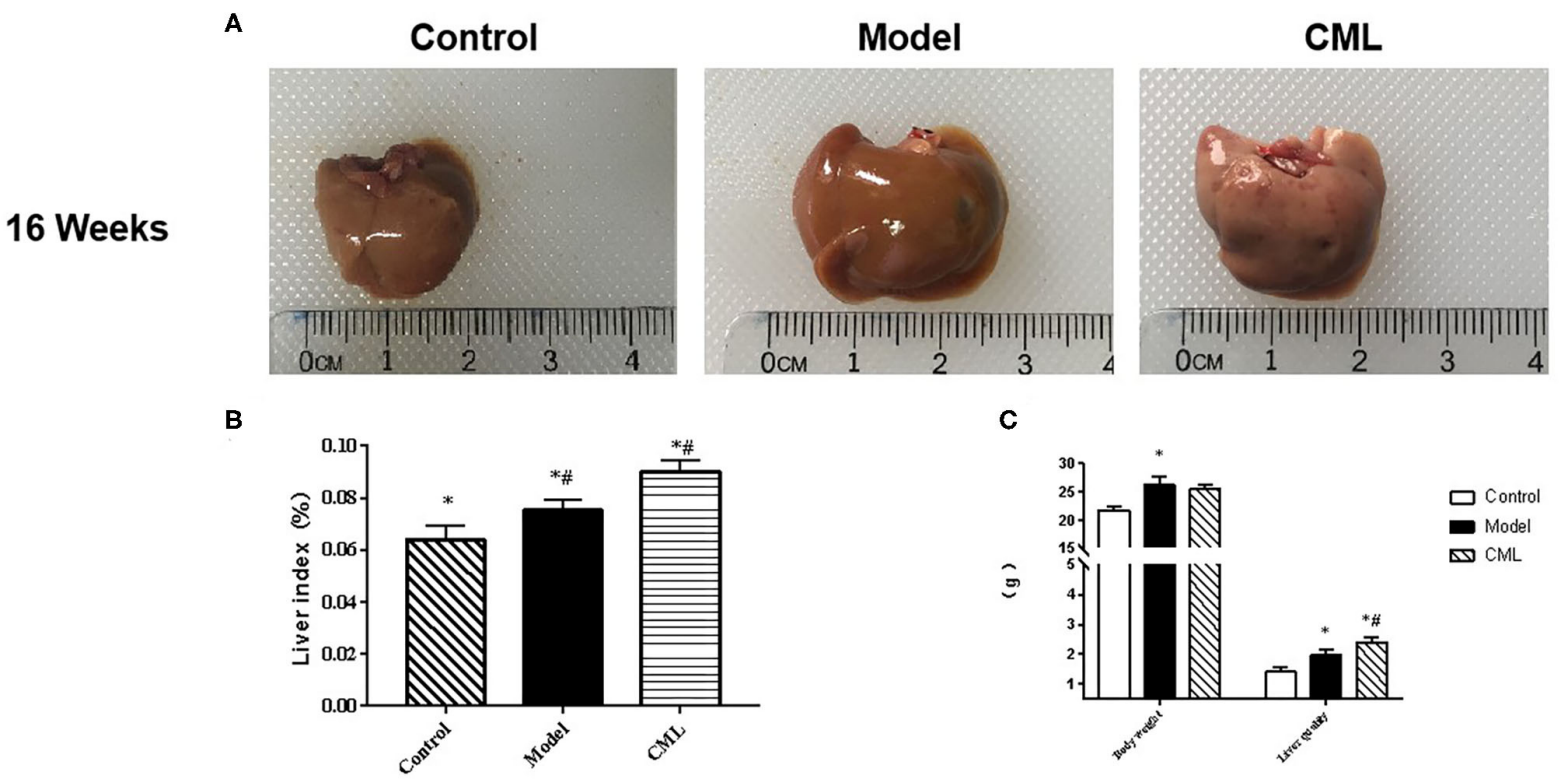
Effect of CML on liver lipid accumulation **(A)**. Observation of overall liver morphology **(B)**. Liver body mass indexin ApoE^−/−^ mice **(C)**. ApoE^−/−^ mice body weight and wet liver weight. *P < 0.05 compared with control group; ^#^*P* < 0.05 compared with model group.

**Figure 4:**
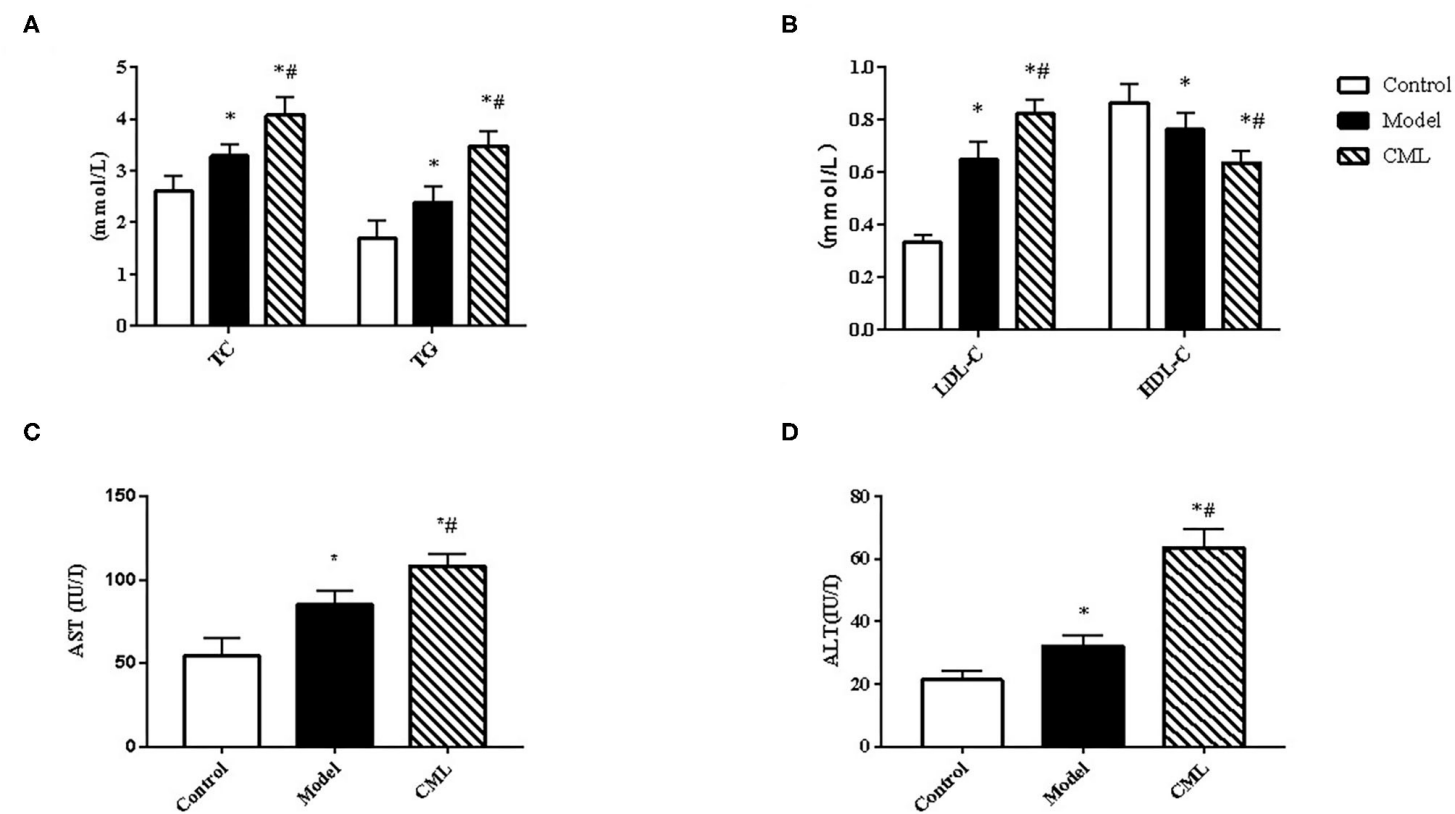
Observe the effect of 16 weeks CML on related biochemical indexes of ApoE^−/−^ mice **(A)**. Total cholesterol (TC), Triglyceride (TG) **(B)**. Low-density lipoprotein (LDL-C) and high-density lipoprotein (HDL-C) **(C)**. Aspartate aminotransferase (AST) **(D)**. Alanine aminotransferase (ALT) levels were measured using an automatic biochemical analyzer. All data are expressed as the means ± SEM. *P < 0.05, compared with control group; ^#^*P* < 0.05, compared with model group.

All ApoE^−/−^ mice were euthanized after 16 weeks. By observing the general picture of mice liver (Figure 3A), we found that compared with the control group, the liver volume of the model group was significantly increased (*P* < 0.05), and the liver surface was greasy. Under CML stimulation, there was no significant change in liver volume (*P* > 0.05), but the liver color was pale, which we speculated may be related to lipid deposition. To prove this idea, we calculate the liver index (Figure 3B). We found that the liver indices of the mice under CML interventions significantly increased compared with the control group (*P* < 0.05), but there was no significant difference in body weights (*P* > 0.05) (Figure 3C). The formation of NAFLD is accompanied by disorders of lipid metabolism and liver damage. Therefore, we measured the levels of TC, TG, LDL-C, HDL-C, AST, and ALT in the serum (Figure 4). After CML intervention, the level of TC, TG, LDL-C, AST, and ALT were significantly higher (*P* < 0.05), But the level of HDL-C is obviously reduced compare with the control group (*P* < 0.05).

**Figure 5:**
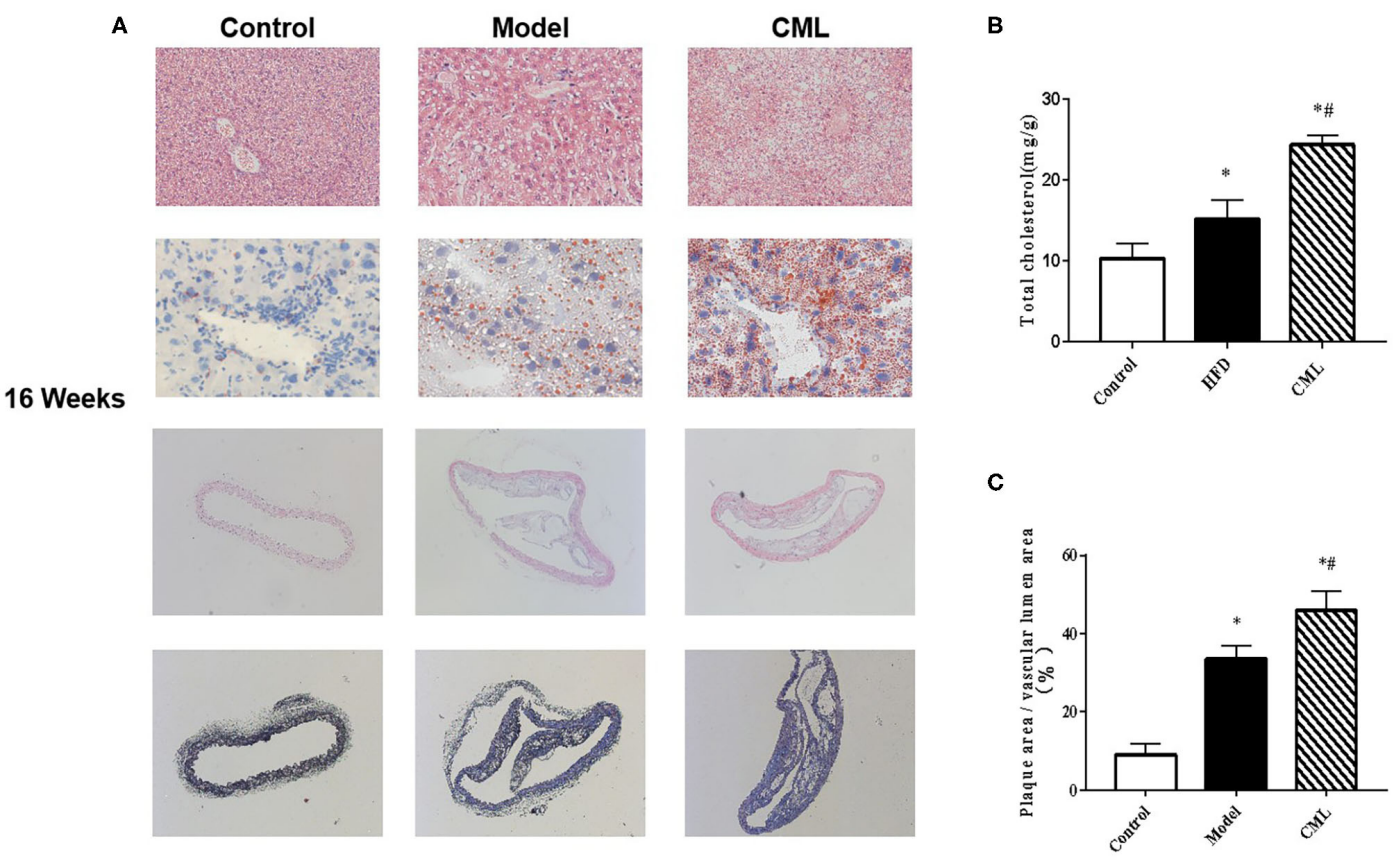
Pathological changes of liver and aorta after ApoE^−/−^ mice at the time of 16 weeks **(A)**. First row: H&E staining of ApoE^−/−^ mice liver (×200). Second row: Oil Red O staining of ApoE^−/−^ mice liver (×400). Third row: H&E staining of ApoE^−/−^ mice aorta (×100). Fourth row: Masson staining of liver aorta in ApoE^−/−^ mice (×100). **(B)**. Total cholesterol content in ApoE^−/−^ mice liver **(C)**. Quantification of vascular plaque area by Image J. *P < 0.05, compared with control group; ^#^*P* < 0.05, compared with model group.

H&E and Oil Red O staining were used to observe the liver pathological changes in the mice (Figures 5A,B). The control group showed normal structures in the liver, without obvious lipid droplet infiltration. After CML intervention, the liver tissue structure was unclear, and typical vacuole-like steatotic cells and red spherical lipid droplets formed. CML effectively induces the formation of NAFLD. Then observe the pathological changes of the aorta by H&E staining and Masson staining (Figures 5A,C). There was no obvious plaque formation in the control group. In the model group showed plaque protruding into the lumen. However, CML group had a significantly larger plaque area in the vascular lumen, a large amount of cholesterol crystals, a weak fiber cap, and poor plaque stability.

### CML Promotes the Expression of Pro-inflammatory Cytokines in the Liver and Blood Vessels of ApoE^-/-^ Mice

**Figure 6:**
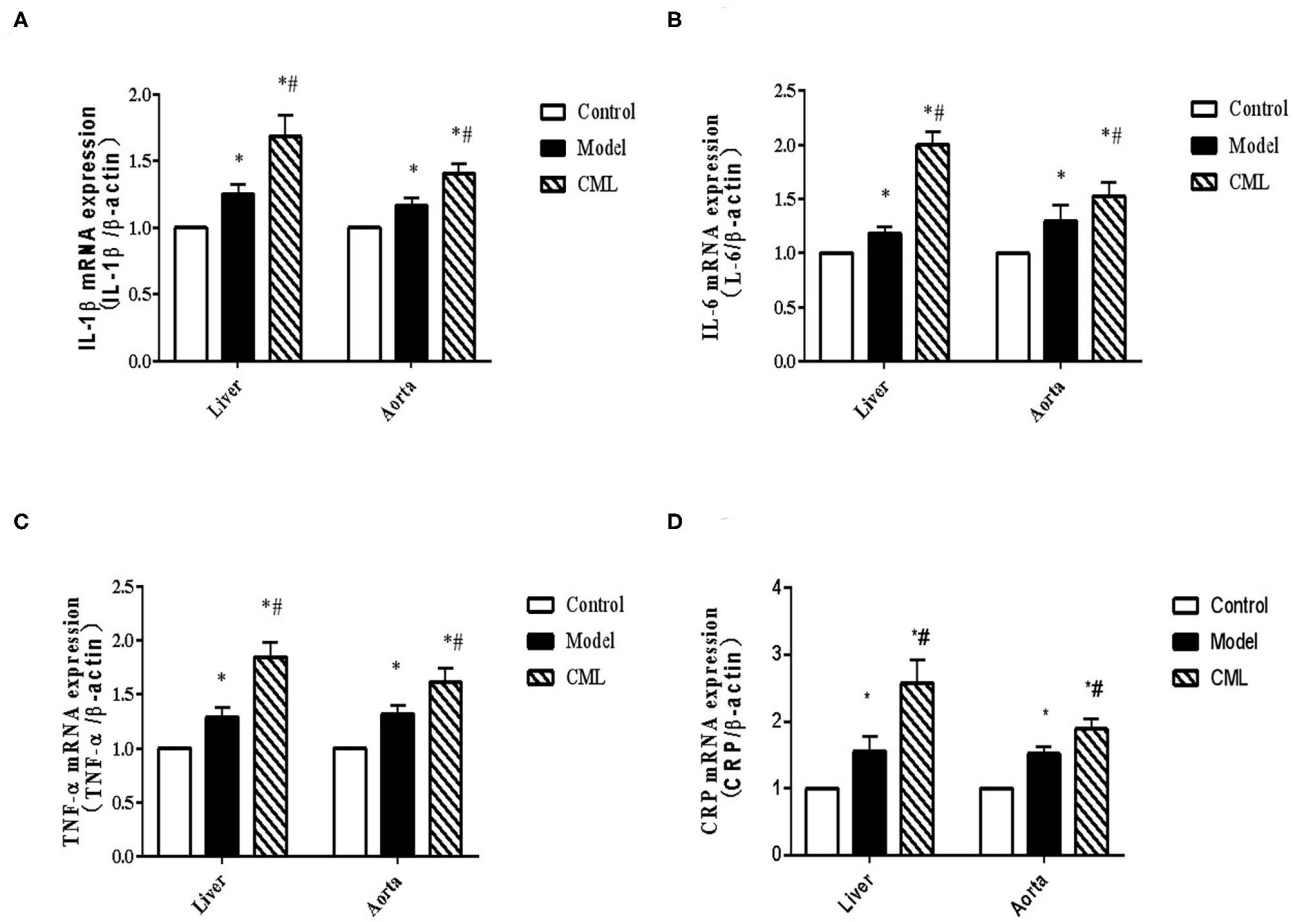
CML promotes the expression of pro-inflammatory cytokines in liver and blood vessels of ApoE^−/−^ mice **(A–D)** Detection of IL-1β, IL-6, TNF-α, CRP mRNA expression levels in liver and aorta by PCR. *P < 0.05, compared with control group; ^#^*P* < 0.05, compared with model group.

To investigate whether the pathogenic role of CML in the liver and aorta is related to inflammation, we examined the expression of IL-1β, IL-6, TNF-α, CRP mRNA in the liver and aorta. We found that IL-1β, IL-6, TNF-α, CRP mRNA expression increased by 1.68, 2.0, 1.85, 2.51 times in liver tissues respectively compared with the control group (Figure 6), while IL-1β, IL-6,TNF-α CRP mRNA expression increased by 1.32, 1.87, 1.19,1.61 times in aorta (Figure 6).

### CML Promotes the Expression of Pro-inflammatory Cytokines by RAGE

**Figure 7:**
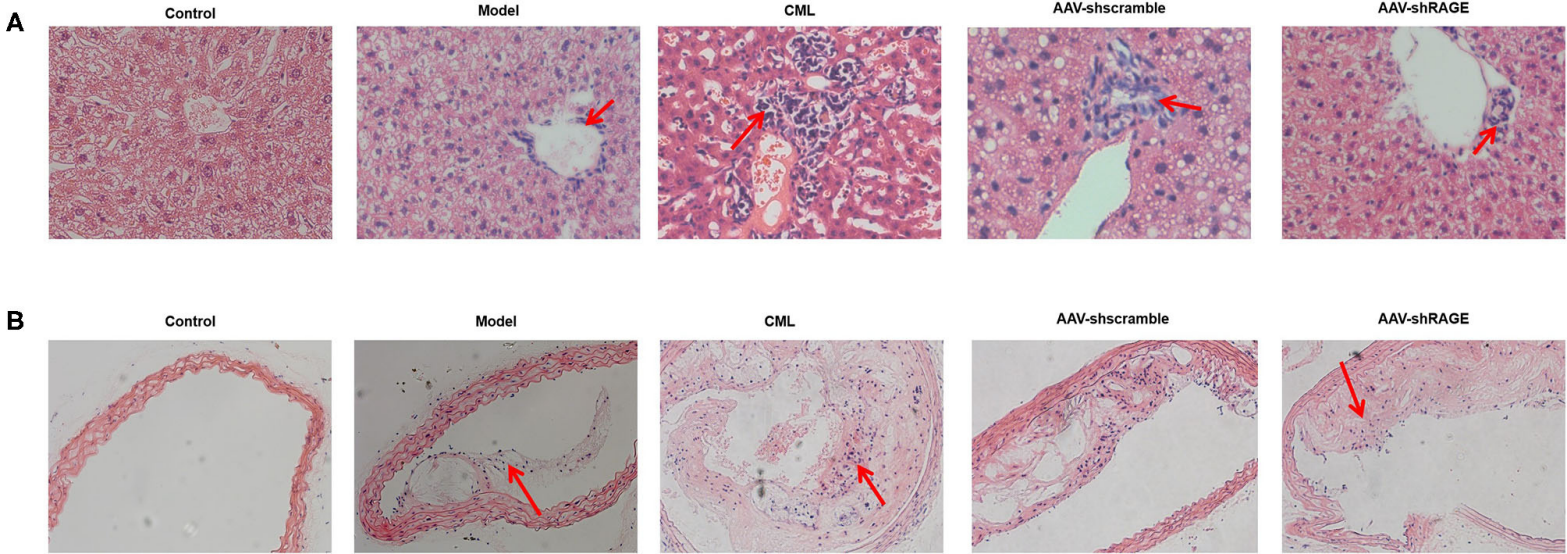
CML increases the density of inflammatory cells infiltrating the plaque and liver **(A)** H&E staining of ApoE^−/−^ mice liver (×200) **(B)** H&E staining of ApoE^−/−^ mice aotra plaque (×200). The red arrow points to the location of inflammatory cell infiltration.

**Figure 8:**
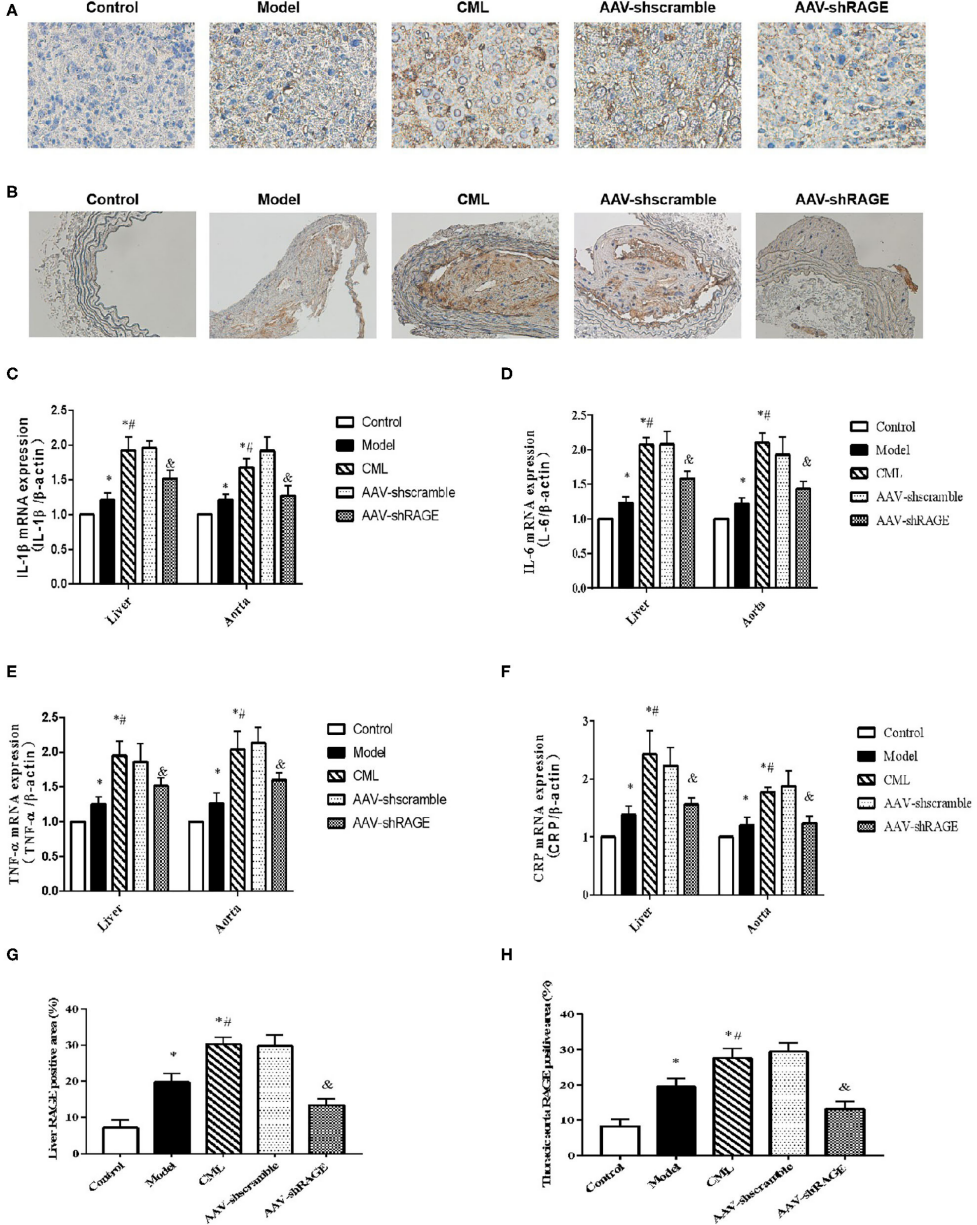
After AAV injection, the expression of RAGE in liver and aorta changed **(A)** and **(B)** Immunohistochemistry shows the expression of RAGE (×400). **(C–F)** Detection of IL-1β, IL-6, TNF-α, CRP mRNA expression levels in liver and aorta by PCR **(G,H)** Percentage of RAGE positive immunohistochemical staining area. The proportion of the positive area to the total Liver area was calculated by ImageJ. *P < 0.05, compared with normal control group; ^#^*P* < 0.05, compared with model group;^&^*P* < 0.05, compared with CML group.

Inflammatory cell infiltration in liver and aorta were observed by H&E staining (Figure 7). There was no significant inflammatory cell infiltration in the control group. In the model group, infiltration of inflammatory cells in liver tissue and aortic plaques can be observed. However, after CML intervention, the inflammatory cell infiltration in the liver tissue and aortic plaques were significantly aggravated, focal necrosis of hepatocytes was observed. After silencing the expression of RAGE, the inflammatory cell infiltration in the liver tissue and aortic plaques were significantly reduced. To detect whether CML promotes the expression of pro-inflammatory cytokines by upregulating the expression of RAGE, we used CML combined with AAV treatment (AAV-shRAGE and AAV-shscramble). Immunohistochemistry and quantification showed that RAGE expression did not change significantly after the injection of AAV-shscramble. AAV-shRAGE significantly downregulated the expression of RAGE (Figures 8A,B,G,H). We measured the expression of IL-1β, IL-6, TNF-α, and CRP mRNA in the liver and aorta (Figures 8C–F). The results showed that the levels of IL-1β, IL-6, TNF-α, and CRP mRNA in liver and aorta were significantly lower in the AAV-shRAGE group compared with the CML group (*P* < 0.05). This may be related to silencing the expression of RAGE down-regulating the expression of pro-inflammatory cytokines.

## Discussion

At present, there is a high prevalence of NAFLD in the world, which has attracted increasing attention. NAFLD includes a series of diseases, from simple fatty liver to non-alcoholic steatohepatitis (NASH), which may develop into liver cirrhosis or even liver cancer (27). Studies have shown that in the past 10 years, NAFLD has been associated with liver related incidence rate or mortality (28, 29). But most of NAFLD deaths are due to AS (30–33). In recent years, researchers have found that NAFLD may be the cause of AS, indicating that the relationship between NAFLD and AS is bidirectional or both diseases are caused by a common pathogenic link (34–37). Therefore, it is very important to find the common pathogenic factors between them.

Abnormal lipid metabolism is the basis for the occurrence of AS. Hyperlipidemia can directly cause endothelial cell dysfunction, increase the permeability of endothelial cells, and provide a basis for lipid deposition on the vascular inner membrane and platelet adhesion (38). Therefore, lipids Metabolic abnormalities are believed to be related to the acceleration of the progression of AS. As early as 1998, Professor ROSS proved that AS is a chronic persistent inflammatory disease (39). The development process of AS can be divided into lipid streak stage, fibrous plaque stage, atheroma stage, unstable plaque stage, plaque rupture and thrombosis stage. In the different development stages of AS, AS is always accompanied by inflammatory reactions (40). IL-1β, CRP, TNF-α, and IL-6 are commonly used indicators to assess the risk of cardiovascular events (41, 42). These pro-inflammatory cytokines are also closely related to the occurrence and prognosis of NAFLD disease (43, 44). Therefore, regulating lipids and inhibiting inflammation have become an important way to combat AS in recent years. Increased fatty liver inflammation is a sign of the progression of NAFLD, therefore, inhibiting inflammation damage is also an important way to prevent and treat NAFLD (45).

Studies have found that NAFLD is formed during the occurrence and development of AS and can promote the development of AS (46). The main mechanism may be that the formation of NAFLD causes the body to be in a chronic inflammatory state for a long time, promotes the release of pro-inflammatory cytokines, leads to damage to the endothelial cells of the vascular intima, and accelerates the formation of atherosclerosis (47–49). The blood lipid metabolism disorder of NAFLD patients is manifested by plasma hypertriglyceridemia, increased low-density lipoprotein and decreased high-density lipoprotein levels, which also contribute to the development of AS (50).

In our clinical study, we found that CML aggregation in liver tissue of NAFLD patients, with the progress of liver steatosis, the expression of CML significantly increased. Therefore, we hypothesized that CML participated in the development of NAFLD, to explore the relationship between CML and non-alcoholic fatty liver and atherosclerosis. We established an *in vivo* model of ApoE^−/−^ mice and monitored the liver and vascular lesions at 4, 8, and 16 weeks respectively. It was found that the fatty degeneration of the liver and the change of the plaque in the aorta gradually increased with the change of time. Moreover, fatty degeneration of liver occurs earlier than atherosclerosis.

NAFLD is a disease characterized by dyslipidemia and impaired liver function (51). Hyperlipidemia is an important risk factor for fatty liver formation. 20–92% of patients with hyperlipidemia have fatty liver (52). NAFLD is not only the excessive deposition of liver fat, but also the damage of liver cell function. AST and ALT in serum were commonly used as indexes to measure the function of hepatocytes (53). After 16 weeks of experimental intervention, serum related biochemical indexes and morphological changes of liver were detected. We found that the serum TG, TC, LDL-C, ALT, AST levels increased and HDL-C levels decreased in the model group after 16 weeks of continuous high fat feeding. The model of mice NAFLD was established successfully, and obvious plaque formation was found in the vascular lumen. After CML stimulation, the lesions of mice non-alcoholic fatty liver were aggravated and the plaque was more unstable. This confirms our hypothesis that CML is the link of NAFLD and AS.

How CML mediates the pathogenesis of NAFLD and AS has attracted our attention. In clinical trials, we found that in the liver samples of NAFLD, RAGE staining was stronger and positively correlated with fatty liver degeneration. Studies have shown that RAGE is an important receptor for CML, which is difficult to clear after binding to its receptor and can activate multiple signal cascades, target genes that regulate the inflammatory response (54). Excessive inflammation is not only a sign of the progression of NAFLD to Non-alcoholic steatohepatitis (NASH) but also an important influencing factor of as progress (55–57). *In vivo* study, we found that after CML stimulation, significant infiltration of inflammatory cells and focal necrosis were observed in liver tissue. This may indicate that CML can promote the progression of NAFLD to NASH.

The expression levels of RAGE and pro-inflammatory cytokines IL-1β, IL-6, TNF-α, CRP in liver and aorta were significantly increased after CML stimulation. In this study, when RAGE was silenced, the expression level of pro-inflammatory cytokines was down regulated, which significantly alleviated the pathological changes of NAFLD and AS. This finding indicates that CML is responsible for the up-regulation of pro-inflammatory cytokines by up-regulating RAGE expression.

In this study, we confirmed that the activation of CML/RAGE signal leads to an imbalance in pro-inflammatory cytokine expression mediates the development of NAFLD and AS. Inhibition of CML/RAGE signal in liver can inhibit the occurrence of NAFLD and delay the progression of AS. It is hoped that our findings could provide a worthwhile intervention target for the treatment and prevention of AS in NAFLD.

## Data Availability Statement

The datasets presented in this study can be found in online repositories. The names of the repository/repositories and accession number(s) can be found in the article/Supplementary Material.

## Ethics Statement

The animal study was reviewed and approved by Institutional Animal Care and Use Committee at Jiangsu University. Human studies conform to the principles outlined in the Declaration of Helsinki (1964) and was approved by the Ethical Committee of the Affiliated Hospital of Jiangsu University. All patients agreed and signed informed consent before enrollment.

## Author Contributions

QP designed experimental ideas, completed experiments, and analyzed data. ZW provided suggestions for experimental design. ZB and ZS assisted the analysis of experimental data. LZ and LJ assisted in collecting clinical data. LL assisted in preparing sections and assessing the degree of liver tissue lesion. CS, HC, and LW supervised the experiment and read and approved the final draft. QP completed the manuscript with the help of everyone. All authors are involved in experimental research.

## Conflict of Interest

The authors declare that the research was conducted in the absence of any commercial or financial relationships that could be construed as a potential conflict of interest.

## References

[1] Zelber-Sagi S Ratziu V Oren R. Nutrition and physical activity in NAFLD: an overview of the epidemiological evidence. World J Gastroenterol. (2011) 17:3377–89. 10.3748/wjg.v17.i29.3377 21876630 PMC3160564

[2] Dongiovanni P Romeo S Valenti L. Genetic factors in the pathogenesis of nonalcoholic fatty liver and steatohepatitis. Biomed Res Int. (2015) 2015:1–10. 10.1155/2015/460190 26273621 PMC4530215

[3] Romero FA Jones CT Xu Y Fenaux M Halcomb RL. The race to bash NASH: emerging targets and drug development in a complex liver disease. J Med Chem. (2020) 63:5031–73. 10.1021/acs.jmedchem.9b01701 31930920

[4] Sherif ZA Saeed A Ghavimi S Nouraie SM Laiyemo AO Brim H. Global epidemiology of non-alcoholic fatty liver disease and perspectives on US minority populations. Dig Dis Sci. (2016) 61:1214–25. 10.1007/s10620-016-4143-0 27038448 PMC4838529

[5] Ismail MH. Nonalcoholic fatty liver disease and type 2 diabetes mellitus: the hidden epidemic. Am J Med Sci Jun. (2011) 341:485–92. 10.1097/MAJ.0b013e3182018598 21412139

[6] Younossi ZM Golabi P de Avila L Paik JM Srishord M Fukui N. The global epidemiology of NAFLD NASH in patients with type 2 diabetes: a systematic review meta-analysis. J Hepatol. (2019) 71:793–801. 10.1016/j.jhep.2019.06.021 31279902

[7] Fracanzani AL Valenti L Bugianesi E Andreoletti M Colli A Vanni E. Risk of severe liver disease in nonalcoholic fatty liver disease with normal aminotransferase levels: a role for insulin resistance and diabetes. Hepatology. (2008) 48:792–8. 10.1002/hep.22429 18752331

[8] Kotronen A Juurinen L Hakkarainen A Westerbacka J Cornér A Bergholm R. Liver fat is increased in type 2 diabetic patients and underestimated by serum alanine aminotransferase compared with equally obese nondiabetic subjects. Diabetes Care. (2008) 31:165–9. 10.2337/dc07-1463 17934148

[9] Gastaldelli A Cusi K Pettiti M Hardies J Miyazaki Y Berria R. Relationship between hepatic/visceral fat and hepatic insulin resistance in nondiabetic and type 2 diabetic subjects. Gastroenterology. (2007) 133:496–506. 10.1053/j.gastro.2007.04.068 17681171

[10] Mantovani A. Nonalcoholic Fatty Liver Disease (NAFLD) and risk of cardiac arrhythmias: a new aspect of the liver-heart axis. J Clin Transl Hepatol. (2017) 5:134–41. 10.14218/JCTH.2017.00005 28660151 PMC5472934

[11] Tana C Ballestri S Ricci F Di Vincenzo A Ticinesi A Gallina S. Cardiovascular risk in non-alcoholic fatty liver disease: mechanisms and therapeutic implications. Int J Environ Res Public Health. (2019) 16:3104–23. 10.3390/ijerph16173104 31455011 PMC6747357

[12] Sookoian S Pirola CJ. Non-alcoholic fatty liver disease is strongly associated with carotid atherosclerosis: a systematic review. J Hepatol. (2008) 49:600–7. 10.1016/j.jhep.2008.06.012 18672311

[13] Day CP James OF. Steatohepatitis: a tale of two “hits”? Gastroenterology. (1998) 114:842–5. 10.1016/S0016-5085(98)70599-2 9547102

[14] Dos Santos JM Tewari S Mendes RH. The role of oxidative stress in the development of diabetes mellitus and its complications. J Diabetes Res. (2019) 2019:189813. 10.1155/2019/4189813 31192263 PMC6525877

[15] Baynes JW Thorpe SR. Role of oxidative stress in diabetic complications: a new perspective on an old paradigm. Diabetes. (1999) 48:1–9. 10.2337/diabetes.48.1.1 9892215

[16] Gaens KH Stehouwer CD Schalkwijk CG. The N^ε^-(carboxymethyl)lysine -RAGE axis: putative implications for the pathogenesis of obesity-related complications. Endocrinol Metab. (2010) 5:839–54. 10.1586/eem.10.68 30780826

[17] Fu MX Requena JR Jenkins AJ Lyons TJ Baynes JW Thorpe SR. The advanced glycation end product, Nepsilon-(carboxymethyl)lysine, is a product of both lipid peroxidation and glycoxidation reactions. J Biol Chem. (1996) 271:9982–6. 10.1074/jbc.271.17.9982 8626637

[18] Wang Z Jiang Y Liu N Ren L Zhu Y An Y. Advanced glycation end-product N^ε^-carboxymethyl-Lysine accelerates progression of atherosclerotic calcification in diabetes. Atherosclerosis. (2012) 221:387–96. 10.1016/j.atherosclerosis.2012.01.019 22305260

[19] Stojsavljević S Gomerčić Palčić M Virović Jukić L Smirčić Duvnjak L Duvnjak M. Adipokines proinflammatory cytokines, the key mediators in the pathogenesis of nonalcoholic fatty liver disease. World J Gastroenterol. (2014) 20:18070–91. 10.3748/wjg.v20.i48.18070 25561778 PMC4277948

[20] Nonaka K Kajiura Y Bando M Sakamoto E Inagaki Y Lew JH. Advanced glycation end-products increase IL-6 and ICAM-1 expression via RAGE, MAPK and NF-κB pathways in human gingival fibroblasts. J Periodontal Res. (2018) 53:334–44. 10.1111/jre.12518 29193068

[21] Liu SH Sheu WH Lee MR Lee WJ Yi YC Yang TJ. Advanced glycation end product N^ε^ - carboxymethyllysine induces endothelial cell injury: the involvement of SHP-1-regulated VEGFR-2 dephosphorylation. Pathology. (2013) 230:215–27. 10.1002/path.4045 22553146

[22] Wood TT Winden DR Marlor DR Wright AJ Jones CM Chavarria M. Acute secondhand smoke-induced pulmonary inflammation is diminished in RAGE knockout mice. Am J Physiol Lung Cell Mol Physiol. (2014) 307:758–64. 10.1152/ajplung.00185.2014 25260756

[23] Wang Z Yan J Li L Liu N Liang Y Yuan W. Effects of N^ε^ -carboxymethyl-lysine on ERS-mediated apoptosis in diabetic atherosclerosis. Int J Cardiol. (2014) 172:478–83. 10.1016/j.ijcard.2014.01.031 24491856

[24] Araki E Nishikawa T. Oxidative stress: a cause and therapeutic target of diabetic complications. J Diabetes Investig. (2010) 1:90–6. 10.1111/j.2040-1124.2010.00013.x 24843413 PMC4008021

[25] Allahverdian S Chehroudi AC McManus BM Abraham T Francis GA. Contribution of intimal smooth muscle cells to cholesterol accumulation and macrophage-like cells in human atherosclerosis. Circulation. (2014) 129:1551–9. 10.1161/CIRCULATIONAHA.113.005015 24481950

[26] Butscheid M Hauptvogel P Fritz P Klotz U Alscher DM. Hepaticex-pressionofgalectin-3 and receptor for advanced glycationend products inpatients with liver disease. J Clin Pathol. (2007) 60:415–8. 10.1136/jcp.2005.032391 16775125 PMC2001117

[27] Oates JR McKell MC Moreno-Fernandez ME Damen MSMA Deepe GS Qualls JE. Macrophage function in the pathogenesis of non-alcoholic fatty liver disease: the mac attack. Front Immunol. (2019) 10:2893–920. 10.3389/fimmu.2019.02893 31921154 PMC6922022

[28] Angulo P Hui JM Marchesini G Bugianesi E George J Farrell GC. The NAFLD fibrosis score: a noninvasive system that identifies liver fibrosis in patients with NAFLD. Hepatology. (2007) 45:846–54. 10.1002/hep.21496 17393509

[29] Chalasani N Younossi Z Lavine JE Diehl AM Brunt EM Cusi K. The diagnosis and management of non-alcoholic fatty liver disease: practice guideline by the American gastroenterological association, American Association for the study of liver diseases, and American College of Gastroenterology. Gastroenterology. (2012) 142:1592–609. 10.1053/j.gastro.2012.04.001 22656328

[30] Ong JP Pitts A Younossi ZM. Increased overall mortality and liver-related mortality in non-alcoholic fatty liver disease. J Hepatol. (2008) 49:608–12. 10.1016/j.jhep.2008.06.018 18682312

[31] Söderberg C Stål P Askling J Glaumann H Lindberg G Marmur J. Decreased survival of subjects with elevated liver function tests during a 28-year follow-up. Hepatology. (2010) 51:595–602. 10.1002/hep.23314 20014114

[32] Lonardo A Targher G. Cardiovascular risk in NAFLD: an intimate relationship? Dig Dis Sci. (2019) 5:595–602. 10.1007/s10620-019-05996-7 31820180

[33] Stepanova M Younossi ZM. Independent association between nonalcoholic fatty liver disease and cardiovascular disease in the US population. Clin Gastroenterol Hepatol. (2012) 10:646–50. 10.1016/j.cgh.2011.12.039 22245962

[34] Targher G Byrne CD Lonardo A Zoppini G Barbui C. Nonalcoholic fatty liver disease and risk of incident cardiovascular disease: a meta-analysis of observational studies. J Hepatol. (2016) 65:589–600. 10.1016/j.jhep.2016.05.013 27212244

[35] Baratta F Pastori D Angelico F Balla A Paganini AM Cocomello N. Nonalcoholic fatty liver disease and fibrosis associated with increased risk of cardiovascular events in a prospective study. Clin Gastroenterol Hepatol. (2020) 18:2324–31.e4. 10.1016/j.cgh.2019.12.026 31887443

[36] Lonardo A Lombardini S Scaglioni F Ballestri S Verrone AM Bertolotti M. Fatty liver, carotid disease and gallstones: a study of age related associations. World J Gastroenterol. (2006) 12:5826–33. 10.3748/wjg.v12.i36.5826 17007049 PMC4100664

[37] Santos RD Valenti L Romeo S. Does nonalcoholic fatty liver disease cause cardiovascular disease? Current knowledge and gaps. Atherosclerosis. (2019) 282:110–20. 10.1016/j.atherosclerosis.2019.01.029 30731283

[38] Novák J Bienertová-Vašku J Kára T Novák M. MicroRNAs involved in the lipid metabolism and their possible implications for atherosclerosis development and treatment. Mediat Inflamm. (2014) 2014:1–14. 10.1155/2014/275867 24876669 PMC4020222

[39] Ross R. Atherosclerosis an inflamrnatory disease. N Engl J Med. (1999) 340:15–26. 10.1056/NEJM199901143400207 9887164

[40] Kim MJ Jung SK. Nutraceuticals for prevention of atherosclerosis: targeting monocyte infiltration to the vascular endotheliu. J Food Biochem. (2020) 446:1–12. 10.1111/jfbc.13200 32189369

[41] Wang J Du A Wang H Li Y. MiR-599 regulates LPS-mediated apoptosis and inflammatory responses through the JAK2/STAT3 signaling pathway via targeting ROCK1 in human umbilical vein endothelial cells. Clin Exp Pharmacol Physiol. (2020) 478:1420–8. 10.1111/1440-1681.13316 32248560

[42] Tuttolomondo A Di Raimondo D Pecoraro R Arnao V Pinto A Licata G Atherosclerosis as an inflammatory disease. Curr Pharm Des. (2012) 18:4266–88. 10.2174/138161212802481237 22390643

[43] Lizardi-Cervera J Chavez-Tapia NC Pérez-Bautista O Ramos MH Uribe M. Association among C-reactive protein, fatty liver disease, and cardiovascular risk. Curr Pharm Des. (2007) 52:2375–9. 10.1007/s10620-006-9262-6 17458697

[44] Zhou Y Dong B Kim KH Choi S Sun Z Wu N. Vitamin D receptor activation in liver macrophages protects against hepatic endoplasmic reticulum stress in mice. Hepatology. (2020) 71:1453–66. 10.1002/hep.30887 31381163

[45] Fabbrini E Sullivan S Klein S. Obesity and nonalcoholic fatty liver disease: biochemical, metabolic, and clinical implications. Hepatology. (2010) 51:679–89. 10.1002/hep.23280 20041406 PMC3575093

[46] Zhang L She ZG Li H Zhang XJ. Non-alcoholic fatty liver disease: a metabolic burden promoting atherosclerosis. Clin Sci. (2020) 134:1775–99. 10.1042/CS20200446 32677680

[47] Srivastava RAK. Life-style-induced metabolic derangement and epigenetic changes promote diabetes and oxidative stress leading to NASH and atherosclerosis severity. J Diabetes Metab Disord. (2018) 17:381–91. 10.1007/s40200-018-0378-y 30918873 PMC6405391

[48] Haukeland JW Damås JK Konopski Z Løberg EM Haaland T Goverud I. Systemic inflammation in nonalcoholic fatty liver disease is characterized by elevated levels of CCL2. Hepatology. (2006) 44:1167–74. 10.1016/j.jhep.2006.02.011 16618517

[49] Lombardi R Fargion S Fracanzani AL. Brain involvement in non-alcoholic fatty liver disease (NAFLD): a systematic review. Dig Liver Dis. (2019) 51:1214–22. 10.1016/j.dld.2019.05.015 31176631

[50] Deprince A Haas JT Staels B. Dysregulated lipid metabolism links NAFLD to cardiovascular disease. Mol Metab. (2020) 30:1–45. 10.1016/j.molmet.2020.101092 33010471 PMC7600388

[51] Bellentani S Scaglioni F Marino M Bedogni G. Epidemiology of non-alcoholic fatty liver disease. Digest Dis. (2010) 28:55–61. 10.1159/000282080 20460905

[52] Cullis PR Chonn A Semple SC. Interactions of liposomes and lipid-based carrier systems with blood proteins: relation to clearance behaviour *in vivo*. Adv Drug Deliver Rev. (1998) 32:3–17. 10.1016/S0169-409X(97)00128-2 10837632

[53] Verma S Jensen D Hart J Mohanty SR. Predictive value of ALT levels for non- alcoholic steatohepatitis (NASH) and advanced fibrosis in non-alcoholic fatty liver disease (NAFLD). Liver Int. (2013) 33:398–405. 10.1111/liv.12226 23763360

[54] Kislinger T Fu C Huber B Qu W Taguchi A Du Yan S. N(epsilon)-(carboxymethyl)lysine adducts of proteins are ligands for receptor for advanced glycation end products that activate cell signaling pathways and modulate gene expression. J Biol Chem. (1999) 274:31740–9. 10.1074/jbc.274.44.31740 10531386

[55] Katsiki N Imprialos K Vlachopoulos C. Editorial: arterial stiffness, central haemodynamics and nonalcoholic fatty liver disease: links with cardiovascular risk and effects of drug treatment. Curr Vasc Pharmacol. (2018) 16:401–4. 10.2174/1570161116666171205105402 29210656

[56] Lonardo A Lugari S Ballestri S Nascimbeni F Baldelli E Maurantonio M. A round trip from nonalcoholic fatty liver disease to diabetes: molecular targets to the rescue? Acta Diabetol. (2019) 56:385–96. 10.1007/s00592-018-1266-0 30519965

[57] Schulz E Anter E Keaney JF. Oxidative stress, antioxidants, and endothelial function. Curr Med Chem. (2004) 11:1093–104. 10.2174/0929867043365369 15134508

