# Supplementary figures and images for: CML/RAGE Signal Bridges a Common Pathogenesis Between Atherosclerosis and Non-alcoholic Fatty Liver
Source: Front Med (Lausanne). 2020 Nov 6;7:583943. doi: 10.3389/fmed.2020.583943 (PMC7677500; doi:10.3389/fmed.2020.583943)

## Slide 1
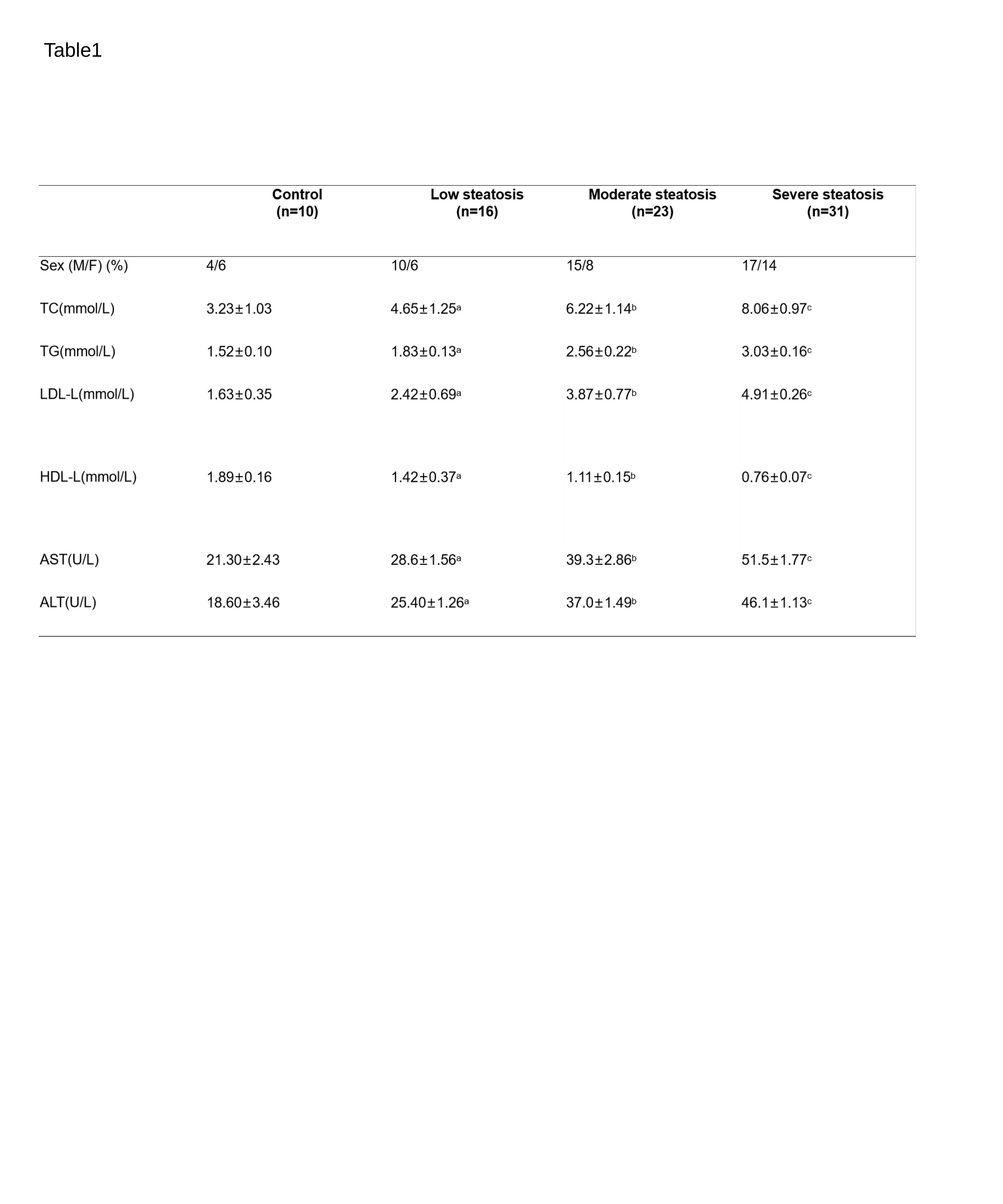

Table1

Supplement: Supplementary file 1 [file Presentation_1.PPTX]
